# Molecular Recognition‐Mediated Transformation of Single‐Chain Polymer Nanoparticles into Crosslinked Polymer Films

**DOI:** 10.1002/anie.201706379

**Published:** 2017-09-08

**Authors:** Clare S. Mahon, Christopher J. McGurk, Scott M. D. Watson, Martin A. Fascione, Chadamas Sakonsinsiri, W. Bruce Turnbull, David A. Fulton

**Affiliations:** ^1^ Chemical Nanoscience Laboratory School of Chemistry Newcastle University Newcastle-upon-Tyne NE1 7RU UK; ^2^ School of Chemistry and Astbury Centre for Structural Molecular Biology University of Leeds Leeds LS2 9JT UK; ^3^ Department of Chemistry University of York Heslington York YO10 5DD UK; ^4^ Department of Biochemistry Faculty of Medicine Khon Kean University Muang District Khon Kaen 40002 Thailand

**Keywords:** adaptive materials, carbohydrates, dynamic covalent chemistry, polymer films, polymer nanoparticles

## Abstract

We describe single‐chain polymer nanoparticles (SCNPs) possessing intramolecular dynamic covalent crosslinks that can transform into polymer films through a molecular recognition‐mediated crosslinking process. The SCNPs utilise molecular recognition with surface‐immobilised proteins to concentrate upon a substrate, bringing the SCNPs into close spatial proximity with one another and allowing their dynamic covalent crosslinkers to undergo intra‐ to interpolymer chain crosslinking leading to the formation of polymeric film. SCNPs must possess both the capacity for specific molecular recognition and a dynamic nature to their intramolecular crosslinkers to form polymer films, and an investigation of the initial phase of film formation indicates it proceeds from features which form upon the surface then grow predominantly in the xy directions. This approach to polymer film formation presents a potential method to “wrap” surfaces displaying molecular recognition motifs—which could potentially include viral, cellular and bacterial surfaces or artificial surfaces displaying multivalent recognition motifs—within a layer of polymer film.

Polymer films are ubiquitous in the modern world, acting as barriers to protect objects from their environments or improve performance.^1^ A plethora of methods exist to prepare polymer films upon surfaces such as solvent casting,^2^ thermal spraying^3^ or by vapour deposition techniques^4^ and self‐assembly methods such as Langmuir–Blodgett^5^ or layer‐by‐layer^6^ approaches. A limitation of these methods is that deposition of polymers upon the surfaces is driven by relatively unselective interactions and additional chemical processes may have to be performed if a crosslinked nature to the coating is desired. Here, we report a method for polymer film formation in which so‐called single‐chain polymer nanoparticles (SCNPs)^7^ are transformed into crosslinked polymeric films. We show that film formation only occurs in the presence of complementary molecular recognition between the SCNPs and functionalities displayed on the surface, and that the dynamic covalent nature of intramolecular crosslinks contained within the SCNPs is crucial for film formation. We anticipate that this method will enable the “wrapping” of a variety of surfaces displaying molecular recognition motifs—which could include the surfaces of viruses, cellular and bacterial surfaces or artificial surfaces displaying multivalent recognition motifs—within a layer of polymer film.

We utilise SCNPs (Figure 1 a, top right) which are nanostructures composed of intramolecularly crosslinked linear polymer chains. We use dynamic covalent acylhydrazone bonds as intramolecular cross‐linkers within our SCNPs on account of the well‐known ability of this bond to undergo component exchange processes in aqueous solution (Figure 1 b).^8^ The dynamic nature of the acylhydrazone linkage endows SCNPs with the capacity for structural reconfiguration, facilitating intra‐ to intermolecular crosslinking of polymer chains. In dilute solution SCNPs possessing dynamic covalent crosslinks have been shown^9^ to display good kinetic stability, but when the SCNPs are concentrated their dynamic crosslinks undergo intra‐ to interchain cross‐linking.9b We anticipated that dynamic covalent SCNPs could be concentrated upon a surface, encouraging their crosslinking to form polymer films. Consequently, our SCNPs are decorated with carbohydrate residues which bind (Figure 1 c) through specific molecular recognition to complementary surface‐immobilised carbohydrate‐binding proteins (lectins). This process concentrates the SCNPs upon the surface where their increased spatial proximity with one another allows intra‐ to inter‐polymer chain reorganization of their dynamic cross‐linkers, leading to formation of polymer film. This topological transformation may be considered as a macromolecular “metamorphosis”.^10^ Galactose and mannose were chosen on account of their specific molecular recognition capabilities with complementary lectins *E. coli* heat labile toxin (LTB) and Concanavalin A (Con A) (Figure 1), respectively. LTB^11^ belongs to the AB_5_ family of toxins^12^ and exhibits recognition behaviour identical to that of cholera toxin, binding to galactose‐terminated gangliosides on cellular surfaces to facilitate entry, with the naturally occurring toxin proceeding to disrupt cellular biochemistry and cause disease. A non‐toxic B_5_ variant of the toxin displaying five circularly arranged galactose binding sites approximately 30 Å apart has been used for this study. Con A^13^ is isolated from *Canavalia ensiformis* (Jack bean), and exists under the conditions of our experiment as a dimer of two 26 kDa subunits which recognises mannose at two binding sites.

**Figure 1:**
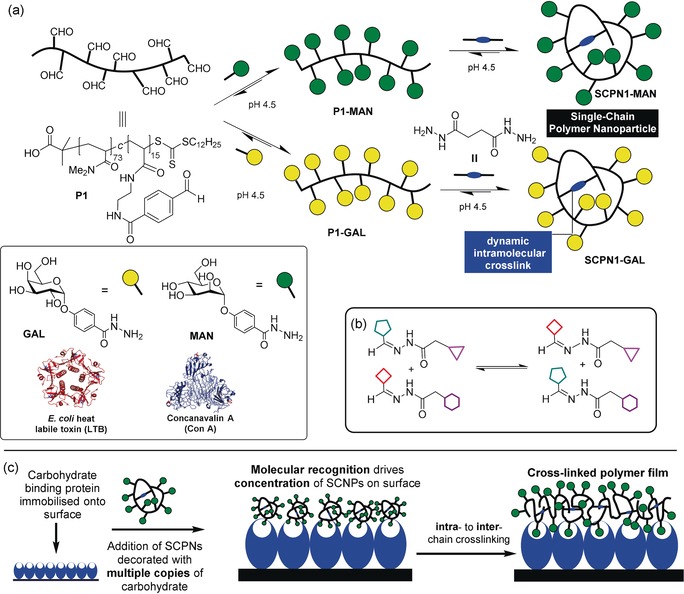
a) Construction of single‐chain polymer nanoparticles (SCNPs): **MAN** and **GAL**, acylhydrazide derivatives of mannose and galactose, were appended onto an aldehyde functionalised polymer scaffold **P1**, producing linear glycopolymers **P1‐MAN** and **P1‐GAL**. Addition of succinic dihydrazide induces intra‐chain crosslinking to yield **SCNP1‐MAN** and **SCNP1‐GAL**. LTB is shown with five associated galactose residues (blue) and Con A shown in its tetrameric form with four associated mannose residues (red). b) Component exchange of dynamic covalent acylhydrazone bonds to form new bonds. c) A combination of specific molecular recognition and rearrangement of dynamic covalent bonds allows SCNPs to cross‐link into polymeric films. Binding to a “Velcro”‐like layer of complementary lectins through specific molecular recognition leads to their concentration upon the surface. The surface‐bound SCNPs are now spatially close, and their intra‐molecular dynamic covalent linkages undergo component exchange to form inter‐chain crosslinks, resulting in formation of polymer film.

SCNPs were prepared from linear polymer scaffold **P1** 14 (Figure 1 a) containing pendant aldehyde functionalities allowing for the conjugation of carbohydrate units14b **GAL** and **MAN** through acylhydrazone linkages, yielding glycopolymers **P1‐GAL** and **P1‐MAN**. Treatment of **P1‐GAL** and **P1‐MAN** with succinic dihydrazide induces component exchange through a transimination‐type process,^15^ resulting in intra‐polymer chain crosslinking to yield the glycosylated SCNPs **SCNP1‐MAN** and **SCNP1‐GAL**. The crosslinking process was monitored by gel permeation chromatography (GPC) which revealed increases in retention time compared to **P1‐GAL** and **P1‐MAN** (Figure S4 in the Supporting Information, SI), an observation which indicates collapse of polymer chains to form species of decreased volume and is consistent with successful SCNP formation.7b, 9a, 16

The kinetic stability of **SCNP1‐MAN** and **SCNP1‐GAL** in solution (9 mg mL^−1^) was monitored by GPC over a 24 h period (Figure S3), during which time no aggregation was observed, indicating SCNPs in solution possess good kinetic stability. Surfaces displaying complementary carbohydrate recognition motifs were prepared by immobilising LTB or Con A onto streptavidin‐coated polystyrene by means of biotin–streptavidin linkages (see the SI). Film formation was performed by immersing the surfaces in solutions (9 mg mL^−1^) of their complementary SCNP (**SCNP1‐GAL** in the case of LTB, and **SCNP1‐MAN** in the case of Con A) for 18 h at 5 °C. Visual inspection suggested the formation of a very thin layer of polymer film which demonstrated no solubility in H_2_O, DMF or DMSO, in contrast to **SCNP1‐MAN** and **SCNP1‐GAL** which possess good solubility in these solvents, observations suggesting the polymer films possessed a crosslinked nature. The polymer films were imaged by AFM (Figure 2). Analysis of the surface treated with **SCNP1‐MAN** (Figure 2 c) revealed feature heights of up to 650 nm and and significant differences in appearance to the Con A‐functionalised substrate (Figure 2 b) and also the underlying streptavidin‐functionalised polystyrene (Figure 2 a), observations consistent with the formation of polymer film.

**Figure 2:**
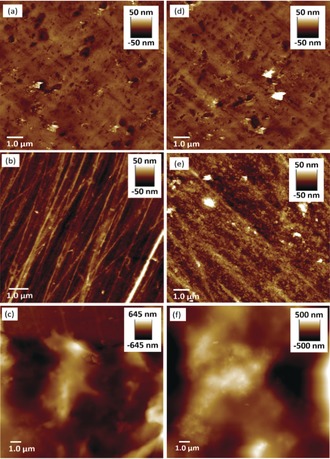
AFM images of a 15.0×15.0 μm region of streptavidin‐coated polystyrene surfaces (a),(d) prior to any modification; b) after functionalisation with Con A; c) after functionalisation with Con A and incubation in a solution of **SCNP1**‐**MAN** for 18 h at 5 °C; e) after functionalisation with LTB via biotin‐streptavidin linkage; f) after functionalisation with LTB and incubation in a solution of **SCNP1**‐**GAL** for 18 h at 5 °C.

Likewise, analysis of the LTB‐functionalised polystyrene after treatment with a solution of **SCNP1‐GAL** (Figure 2 f) indicated the formation of film displaying feature heights up to 500 nm and differences in appearance to the underlying LTB‐functionalised (Figure 2 e) and streptavidin‐functionalised polystyrene (Figure 2 d). Control experiments were performed to gain further insights as to the nature of these polymer films. No significant changes in the nature of streptavidin‐functionalised polystyrene surfaces incubated in solutions of **SCNP1‐MAN** and **SCNP1‐GAL** were observed by AFM (Figure S4a,b). Likewise, no film formation was observed when each lectin‐functionalised polystyrene sample was immersed in a solution of the non‐complementary SCNP, that is, Con A‐functionalised polystyrene with **SCNP1‐GAL**, or LTB‐functionalised polystyrene with **SCNP1‐MAN** (Figure S4c,d). These observations demonstrate that films are only formed when the SCNP displays complementary carbohydrates for the immobilised lectin and that in the absence of specific molecular recognition films do not form. Samples of **SCNP1‐MAN** and **SCNP1‐GAL** were then treated with NaCNBH_3_ to reduce their acylhydrazone bonds thus rendering them unable to undergo intra‐ to interpolymer reconfiguration. Exposure of these “static” SCNPs to complementary lectin‐functionalised polystyrene did not lead to notable changes in appearance of surfaces as determined by AFM (Figure S4e,f), demonstrating that dynamic covalent exchange processes are required for film formation. These observations also indicate that the films are not simply a collection of intramolecularly cross‐linked SCNPs deposited upon a surface, and that reorganization has indeed occurred to afford intermolecularly cross‐linked polymers. Taken together, these control experiments demonstrate that both molecular recognition and dynamic covalent crosslinking are required to drive the transformation of SCNPs into cross‐linked films, and that in the absence of either feature film formation does not occur.

To further support this hypothesis, we exposed pre‐formed polymer films to conditions that would remove the molecular recognition and/or crosslinks. Films of **SCNP1**‐**MAN** on Con A‐functionalised polystyrene (Figure 3 a,c) were incubated in a solution of hydroxylamine to exchange acylhydrazone bonds into more stable oxime bonds^17^ thus releasing the succinic dihydrazide crosslinker along with **MAN**. After 18 h at 5 °C optical microscopy (Figure S5) revealed surfaces free from film. AFM imaging (Figure 3 b) revealed surfaces identical to those observed prior to film formation, suggesting the removal of the cross‐links has led to the disassembly of the polymeric film. This finding demonstrates that acylhydrazone linkages are essential to maintain the structure of films, and that films maintain the dynamic character of their crosslinks. A control experiment was also performed to investigate the importance of the carbohydrate–protein interactions in adhering films to the surfaces. Thus, polymer films prepared from **SCNP1‐MAN** on Con A functionalised polystyrene were incubated at 5 °C for a total of 3 days in a buffered saturated methyl α‐mannoside (a ligand for Con A) solution at pH 4.5. After incubation, microscopy revealed the absence of film, with AFM again showing a surface (Figure 3 d) similar in appearance to that observed prior to film formation. These results demonstrate that undermining molecular recognition processes triggers release of films from surfaces. We hypothesise that the longer timescale required for the removal of films following exposure to methyl α‐mannoside arises because of difficulties in the diffusion of the sugar through the film to reach the recognition sites of Con A units upon the surface. A pre‐formed polymer film was also subjected to mechanical damage (see SI) by using an AFM tip to scratch the surface. After incubation in 100 mm NH_4_OAc (pH 4.5) for 24 h, AFM imaging showed repair of the damaged area, again highlighting the dynamic nature of the material.

**Figure 3:**
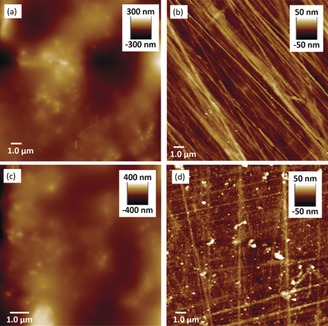
AFM imaging of 15.0×15.0 μm regions of surfaces displaying: a) polymer film produced by exposure of **SCNP1‐MAN** to a Con A functionalised surface; b) the same surface after exposure to hydroxylamine; c) polymer film produced by exposure of **SCNP1‐MAN** to a Con A functionalised surface; d) the same surface after exposure to methyl α‐mannoside.

We then investigated film formation upon lectin‐functionalised silicon wafer, through the formation of a self assembled monolayer (SAM) on the Si surface to which lectins are covalently attached (see SI). This substrate is considerably flatter and smoother than polystyrene, and a surface‐masked plasma etching process (see the SI) can be performed for the removal of organic material from areas of the substrate, allowing film thickness to be determined by AFM. The polymer film obtained from treatment of Con A‐functionalised silicon with **SCNP1‐MAN** for 18 h displays (Figure 4 b) a surface topology which is only subtly different from the underlying Con A‐functionalised silicon (Figure 4 a). The “mask‐etch” procedure was performed and AFM (Figure 4 c) revealed an even film thickness of mean height 17 nm (*σ* 7 nm) (Figure 4 d). The immobilisation of Con A onto the surface through the formation of a SAM and subsequent modification with the lectin is expected to account for ca. 5 nm of organic matter upon the surface,^18^ suggesting a polymer film thickness of around 10 nm. This observation contrasts polymer films obtained upon polystyrene whose feature heights suggest films significantly thicker than those obtained upon Con A‐functionalised silicon. The reasons for this difference are currently unclear; we consider it likely that lectin attachment via organic films onto silicon wafer leads to a more uniform display of protein, providing the foundation for more uniform and thin film formation. Commercially sourced streptavidin‐functionalised polystyrene substrates, on the other hand, may display inhomogeneous lectin coverage which leads to less uniform and and thicker films.

**Figure 4:**
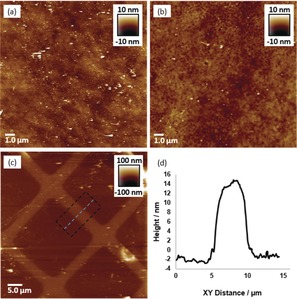
AFM images of a) Con A‐functionalised Si; b) polymer film obtained upon Con A‐functionalised Si; c) the “mask‐etched” polymer film. d) A height profile of the etched polymer film.

To gain insights to the growth of films, multiple samples of Con A‐functionalised silicon wafer were treated in solutions of **SCNP1‐MAN** and wafers removed at time points, rinsed and analysed by AFM. Comparison of the sample at 5 min (Figure 5 b) with the untreated Con A‐functionalised substrate (Figure 5 a) reveals a relatively flat surface upon which has formed a population of features that are 5–10 nm in height and 200–400 nm in diameter (circled in purple). We postulate that these features have formed when multiple SCNPs bind upon areas of the surface where there is an “optimal” display in terms of densities, spacings and orientations of carbohydrate binding sites that promote SCNP concentration and subsequent cross‐linking. There is also a smaller population of larger irregular features (circled in green) of heights 15–30 nm and 0.4–1.0 *μm* in diameter, and we speculate these features occur when two (or more) of the smaller features are sufficiently close together that they merge, or are smaller features that have grown by sequestering and incorporating SCNPs from solution. Analysis of the image obtained at 60 min (Figure 5 c) reveals a greater number of the larger irregular features and that these features have increased their average areas, with fewer of the smaller features. At 360 min (Figure 5 d) the surface was dominated by larger irregular features and even fewer of the smaller features were present. Taken together, these observations indicate that film formation does not occur through a layer‐by‐layer approach where a continuous thin layer is formed which then grows in thickness. Instead, the film is formed in a process where SCNPs concentrate and crosslink at “hotspots” on the surface to form features which then grow predominately in the *xy* plane in an irregular fashion to form larger features. We suggest that feature growth occurs when a SCNP binds to the surface adjacent to a feature and then becomes covalently “trapped” within the feature. Feature growth continues, and in the latter phase of film formation we presume the features merge into a continuous film. A full understanding of the mechanism by which the SCNPs form films is beyond the scope of this work and will be the subject of future experiments.

**Figure 5:**
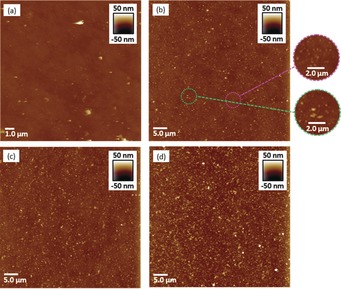
AFM images of a) Con A‐functionalised Si; b) polymer film obtained upon Con A‐functionalised Si at 5 min; c) polymer film obtained upon Con A‐functionalised Si at 60 min; d) polymer film obtained upon Con A‐functionalised Si at 360 min.

The mechanical properties of the films were investigated by AFM nanoindentation analysis. The Young's moduli for polymer surfaces (Table 1) were similar in magnitude to those obtained for plastics such as LDPE or poly(vinyl alcohol),^19^ demonstrating that rearrangement of SCNPs forms a “plastic‐like” coating. Films generated on lectin‐functionalised silicon wafer displayed greater Young's moduli than those corresponding films generated on polystyrene, an observation we attribute to the more uniform lectin coverage expected upon silicon leading to the formation of more homogenous films. Films generated using Con A‐functionalised surfaces and **SCNP1‐MAN** displayed higher Young's moduli than those generated using LTB and **SCNP1‐GAL**, a factor which may complement our visual observation that more extensive film formation was noted on Con A‐functionalised surfaces than on LTB‐functionalised surfaces. We believe that these observations can be accounted for by considering differences in the orientations of binding sites in each lectin (Figure 1). The binding sites of LTB are circularly arranged on one face of the pentamer, whilst the binding sites of Con A point in opposing directions. We propose that the remote geometry of recognition sites in Con A may be better suited to facilitate the formation of more extensive films, and that the resultant films may display improved homogeneity in crosslinking, leading to stiffer materials.

**Table 1:** Mechanical properties of polymer films, as determined by nanoindentation analysis. In each case a minimum of 200 measurements were made, with standard deviations quoted in parentheses.

Substrate	SCNP	Young's modulus of film [GPa]
Si‐Con A	**SCNP1‐MAN**	1.34 (0.521)
Si‐LTB	**SCNP1‐GAL**	0.458 (0.091)
Polystyrene‐Con A	**SCNP1‐MAN**	0.847 (0.309)
Polystyrene‐LTB	**SCNP1‐GAL**	0.131 (0.041)

In conclusion, we have demonstrated how a combination of molecular recognition and dynamic covalent chemistry can be used to drive the transformation of SCNPs into cross‐linked polymeric films. In this work we have utilised recognition between carbohydrate residues and lectins, however, in principle a variety of well‐understood and highly selective molecular recognition motifs could be applied, increasing substantially the scope of the concept. We also propose that the concept could be extended to form films around 3‐dimensional objects displaying high densities of receptors, such as bacteria or viral capsids, thus allowing the application of a “wrapping” of polymer coating which may help to sequester pathogens or possibly even protect and stabilise biological objects. To explore this idea we are investigating the “shrink‐wrapping” of virus‐like particles.

*Dedicated to Sir Fraser Stoddart as he celebrates 50 years of his independent research career*

## Conflict of interest

The authors declare no conflict of interest.

## Supporting information

As a service to our authors and readers, this journal provides supporting information supplied by the authors. Such materials are peer reviewed and may be re‐organized for online delivery, but are not copy‐edited or typeset. Technical support issues arising from supporting information (other than missing files) should be addressed to the authors.

Supplementary
