# Supplementary material for: Molecular Recognition‐Mediated Transformation of Single‐Chain Polymer Nanoparticles into Crosslinked Polymer Films
Source: Angew Chem Int Ed Engl. 2017 Sep 8;56(42):12913–8. doi: 10.1002/anie.201706379 (PMC5656938; doi:10.1002/anie.201706379)
Supplement: Supplementary file 1 — Supplementary [file ANIE-56-12913-s001.pdf]

Angewandte Chemie
Eine Zeitschrift der Gesellschaft Deutscher Chemiker

Supporting Information

Molecular Recognition-Mediated Transformation of Single-Chain Polymer Nanoparticles into Crosslinked Polymer Films

Clare S. Mahon, Christopher J. McGurk, Scott M. D. Watson, Martin A. Fascione, Chadamas Sakonsinsiri, W. Bruce Turnbull, and David A. Fulton**

anie_201706379_sm_miscellaneous_information.pdf

All reagents were purchases from Sigma Aldrich or Alfa Aesar and used as received unless otherwise indicated. Aldehyde monomer **M1**, polymer scaffold **P1** and acylhydrazides **GAL** and **MAN** were prepared according to our previously reported procedures.[1] Con A and biotinylated Con A were supplied by Sigma Aldrich.

Gel permeation chromatography (GPC) was conducted on a Varian ProStar instrument (Varian Inc.) equipped with a Varian 325 UV-Vis dual wavelength detector (254 nm), a Dawn Heleos II multi-angle laser light scattering detector (Wyatt Technology Corp.), a Viscotek 3580 differential RI detector, and a pair of PL gel 5 µm Mixed D 300 × 7.5 mm columns with guard column (Polymer Laboratories Inc.) in series. Near monodisperse methyl methacrylate standards (Agilent Technologies) were used for calibration. Data collection was performed with Galaxie software (Varian Inc.) and chromatograms analyzed with the Cirrus software (Varian Inc.) and Astra software (Wyatt Technology Corp.).Si surfaces were cleaned prior to modification using a Diener Femto plasma asher (100 W, 12 cm^2 min^{-1} O_2, 1 min). Surface topography and nanoindentation data was collected using TappingMode™ atomic force microscopy (AFM), performed in air, on Multimode Nanoscope IIIa and Dimension Nanoscope V systems (Veeco Instruments Inc.) using etched Si probes with resonance frequency 300 kHz. Data acquisition was carried out using Nanoscope version 7.00b19 s on the Dimension Nanoscope V and Nanoscope version 5.12b36 on the Multimode Nanoscope IIIa (Veeco Instruments Inc., Digital Instruments). Data analysis was carried out using Nanoscope Analysis version 1.40 (Bruker).

Experimental Procedures

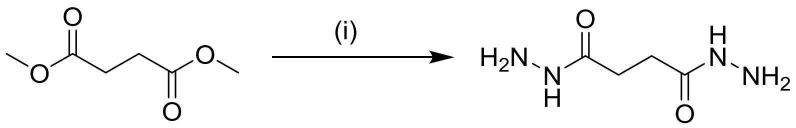

Scheme 1 Preparation of succinic dihydrazide. (i) $N_2H_4.H_2O$, MeOH.

Succinic dihydrazide

Dimethyl succinate (1.00 g, 6.84 mmol, 1 eq.) and hydrazine hydrate (6.64 mL, 137 mmol, 20 eq.) were combined in MeOH (10 mL) and allowed to stir at room temperature, yielding a white precipitate which was isolated by filtration. The product was washed with MeOH and dried under high vacuum (0.69 g, 69%). ^{1}H NMR (500 MHz, dmso-d_6) δ 8.97 (s, 2H, N*H*NH_2), 4.14 (s, 4H, NHN*H_2*), 2.25 (s, 4H (C*H_2*$)_2$) ^{13}C NMR (125 MHz, dmso-d_6) δ 170.86 (CONHNH_2), 28.91 ((C$H_2$$)_2$) Melting point: 167-169 °C.

Procedure for preparation of SCPNs

P1 (15.4 mg, 1.37 µmol, 1 eq.) and **GAL** or **MAN** (7.8 mg, 25 µmol, 18 eq.) were combined in MeOH-d_4 (0.25 mL) and dmso-d_6 (0.25 mL) before addition of 100 mM NH_4OAc, pH 4.5, D_2O (1.0 mL). The solution was left to stir at room temperature until ^1H NMR spectroscopic analysis confirmed complete functionalisation of the polymer, determined by total disappearance of the signal corresponding to the aldehyde proton. A 100 µL aliquot was removed and dried under high vacuum for GPC analysis, before addition of succinic dihydrazide (100 µL from 2.0 mg mL^{-1} stock solution in 100 mM NH_4OAc, pH 4.5, D_2O, 1.37 µmol, 1 eq.). The reaction mixture was allowed to stir at room temperature overnight, before a further 100 µL aliquot was removed and dried under high vacuum for GPC analysis, which confirmed intrachain crosslinking with increase in retention time indicative of a reduction in hydrodynamic volume, consistent with SCPN formation.

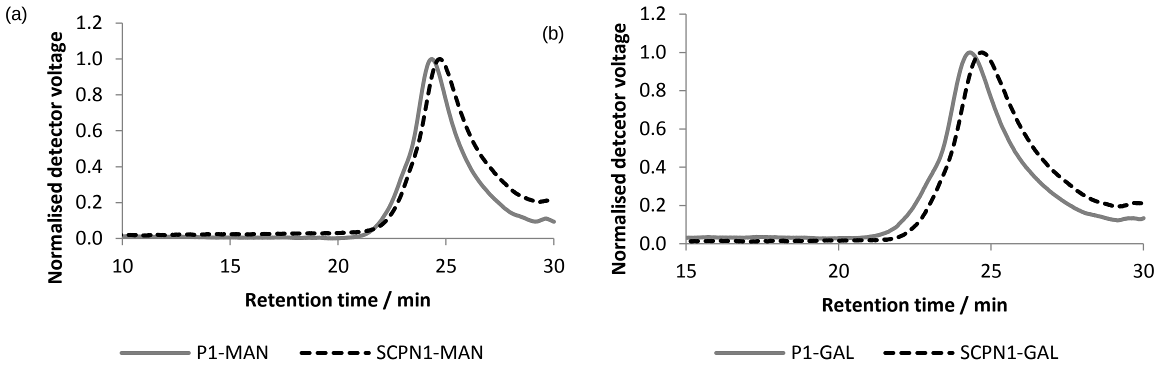

Fig. S1 Differential refractive index gel permeation chromatography (GPC) traces of (a) **P1-MAN** and **SCNP1-MAN** and (b) **P1-GAL** and **SCNP1-GAL** in DMF (0.6 mL/min, containing 1.0 g/L LiBr)

Increasing crosslinking density during SCPN formation

In order to investigate the relationship between the amount of succinic dihydrazide used to crosslink polymer chains and the resultant contraction in hydrodynamic radii during SCPN formation, **P1** was functionalised with **MAN** as described above, yielding glycopolymer **P1-MAN**, and divided into aliquots to which various molar equivalents of succinic dihydrazide were added. These solutions were dried under high vacuum and subjected to GPC analysis (Fig. S2). Increasing the amount of succinic dihydrazide added to **P1-MAN** was shown to increase the retention time of the resultant SCPN for crosslinking densities between 1-3 eq., suggesting that the hydrodynamic volume of the SCPNs has been reduced. Crosslinking with greater molar equivalents of succinic dihydrazide, however, led to the formation of inter-chain crosslinked species, as evidenced by the appearance of a shoulder at lower retention time in their GPC chromatograms.

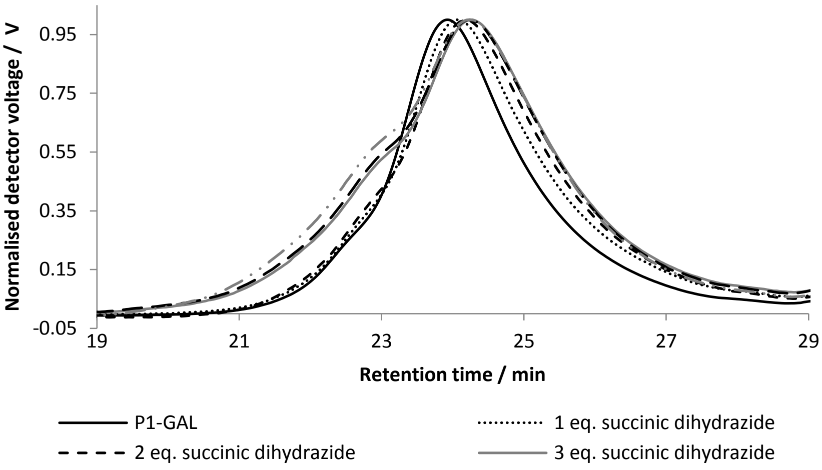

Fig. S2 GPC dRI traces of glycosylated polymer **GP1** and preparations of SCPNs with increasing succinic dihydrazide concentration. Appearance of a shoulder at lower retention time (> 3 eq. succinic dihydrazide) suggests the formation of larger molecular aggregates associated with inter-chain crosslinking.

SCPN stability in solution

A solution of **SCPN1-MAN**, prepared as described earlier, was left to stir at room temperature. 100 µL aliquots were removed at timed intervals over a 24 h period, dried under vacuum and subjected to GPC analysis (Fig. S3). GPC analysis demonstrated that there was no significant aggregation to form larger macromolecular species over this time period.

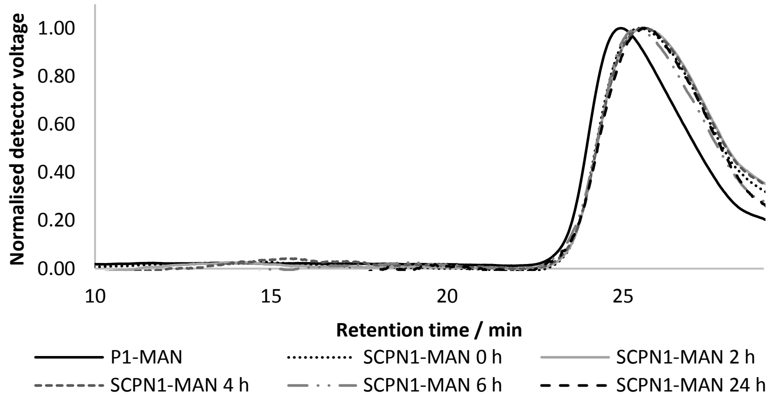

Fig. S3 GPC dRI traces of mannosyl-functionalised polymer **P1-MAN** and a solution of **SCPN1-MAN** over a 24 h period.

Expression of E. coli heat labile toxin (LTB)

Cells from a glycerol stock of *Vibrio sp60* harbouring plasmid pMMB68 (kindly provided by Prof. Tim Hirst)[2] were used to inoculate growth medium (100 mL, 25 g/L LB mix, 15 g/L NaCl, ampicillin 100 µg/mL). The culture was grown overnight at 30 °C with shaking at 200 rpm, then used to inoculate fresh growth medium (6 x 1 L, 25 g/L LB mix, 15 g/L NaCl, ampicillin 100 µg/mL). These cultures were incubated at 30 °C with shaking at 200 rpm until A_{600} reached 0.6 before the protein expression was induced by addition of isopropyl β-D-1-thiogalactopyranoside to a concentration of 0.5 mM. Cultures were incubated (30 °C, 200 rpm) for a further 24 h, then cells were removed by centrifugation (6500 rpm, 15 min). The combined supernatant was treated with ammonium sulphate (550 g/L) and left to stir at 5 °C overnight. Crude protein was isolated by centrifugation (10,000 rpm, 25 min) and redissolved in in 100 mM NaH_2PO_4, pH 7.0, 500 mM NaCl (60 mL). Insoluble material was removed by centrifugation (10,000 rpm, 10 min) before the solution was passed through a 0.22 µm filter then loaded onto a lactose-sepharose 6B column and eluted with 300 mM lactose, 100 mM NaH_2PO_4, pH 7.0, 500 mM NaCl. LTB was dialysed against PBS, pH 7.4, freeze-dried and stored at -80 °C

Preparation of biotinyl-LTB

LTB (900 µL, 647 µM protomer concentration) in PBS was mixed with Pierce EZ-link™ Sulfo-NHS-SS-Biotin (3.5 mg dissolved in 500 µL water). After 2 hours at room temperature, the solution was diluted (PBS, 15 mL) and concentrated by centrifugal ultrafiltration (10kDa MW/CO) twice (final volume 2 mL) before freeze-drying in 5 aliquots and storing at -80 °C until required.

Procedure for immobilisation of lectins onto polystyrene surfaces

The bases of Pierce™ streptavidin coated high capacity 96 well plates were extracted with a cork borer and washed with H_2O before immersion in a solution of the appropriate biotinylated lectin (1.3 mg mL^{-1}, H_2O) for 1 h at 5 °C. The discs were then washed (H_2O) and dried carefully under laminar flow before imaging, or immersion in solutions of SCPNs to prepare polymer films.

Procedure for *N*-hydroxysuccinimide functionalisation of Si wafer[3]

Silicon substrates were cut from a single wafer (100, p-doped) and cleaned by sonication in acetone (15 min), H_2O (15 min) and EtOH (15 min). Surfaces were then treated with O_2 plasma (15 min, 90 W). Substrates were immersed in 2 % v/v (3-aminopropyl)triethoxysilane (APTES) in anhydrous toluene for 45 min, removed an sonicated in toluene (15 min) followed by fresh toluene (15 min) and annealed at 150 °C for 2 h. Substrates were sonicated in H_2O (15 min) and dried under a gentle stream of N_2. APTES functionalised surfaces were immersed in THF containing succinic anhydride (5 mg mL^{-1}) and Et_3N (5 % v/v) for 4 h. Substrates were removed and sonicated in H_2O (2 x 15 min) before drying under a gentle stream of N_2. Surfaces were then immersed in a mixture of 1-ethyl-3-(3-dimethylaminopropyl)carbodiimide (EDC) (50 mg mL^{-1}), *N*-hydroxysuccinimide (NHS) (5 mg mL^{-1}) in 0.5 M 2-(N-morpholino)ethanesulfonic acid (MES) for 3 h. Substrates were removed and sonicated in H_2O (2 x 15 min) and dried under a gentle stream of N_2. NHS functionalised Si substrates were stored at 5 °C.

Procedure for immobilisation of lectins onto *N*-hydroxysuccinimide functionalised Si wafer[3]

NHS-functionalised Si wafers were immersed in a solution of Con A or LTB (0.02 mg mL^{-1}) in phosphate buffered saline (PBS) at pH 7.4 for 5 h. Substrates were then removed and sonicated in fresh PBS (2 x 15 min), then rinsed with H_2O. Surfaces were then immersed in 0.1 M aqueous ethanolamine solution for 1 h, rinsed with H_2O and dried under a gentle stream of N_2.

Procedure for plasma lithography of films

Electron microscopy grids (1000 mesh x 25 µm pitch, copper, Sigma Aldrich) were affixed to surfaces upon which polymer films had been formed on Si wafer as described above using copper adhesive tape (RS components). The surfaces were treated with oxygen plasma (100 W, 12 cm^3 min^{-1}, 5 min) and imaged using Tapping Mode™ AFM.

AFM images for control experiments

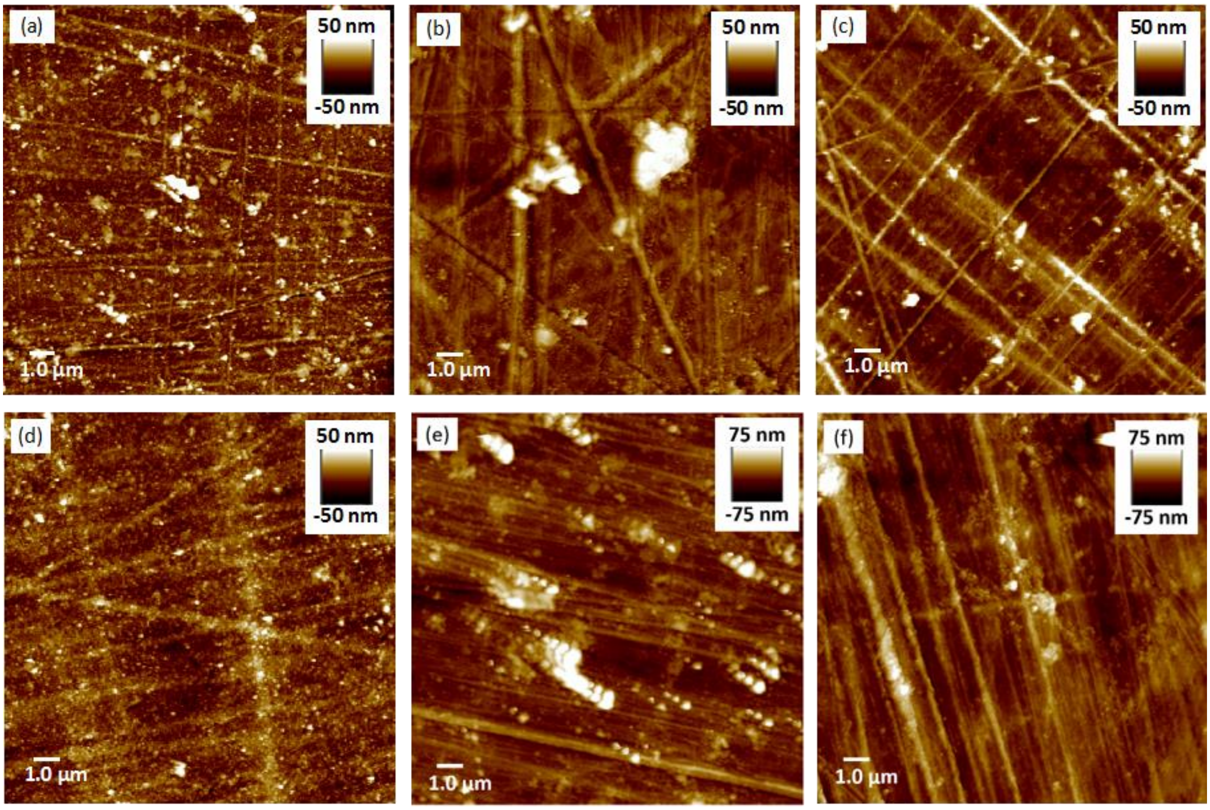

Fig. S4 Tapping Mode™ AFM images of 15.0 x 15.0 µm regions of (a) a streptavidin functionalised surface incubated for 18 h at 5°C in a solution of **SCPN1-MAN**; (b) a streptavidin functionalised surface incubated for 18 h at 5°C in a solution of **SCPN1-GAL**; (c) a Con A functionalised polystyrene surface incubated for 18 h at 5°C in a solution of **SCPN1-GAL**; (d) an LTB functionalised polystyrene surface incubated for 18 h at 5°C in a solution of **SCPN1-MAN**; (e) a Con A functionalised polystyrene surface incubated for 18 h at 5°C in a solution of **SCPN1-MAN** which had been reduced by treatment with $NaCNBH_3$; (f) an LTB functionalised polystyrene surface incubated for 18 h at 5°C in a solution of **SCPN1-GAL** which had been reduced by treatment with $NaCNBH_3$.

Film reversal experiments

Polymer films of **SCPN1-MAN** on Con A functionalised polystyrene were prepared as earlier described and subjected to AFM analysis. Substrates were then incubated at 5 °C in (a) a 50 % v/v solution of $NH_2OH_{(aq)}$, or (b) a saturated solution of methyl α-mannoside in 100 mM NH_4OAc, pH 4.5. Samples were removed after 18 h and the surfaces were examined by optical microscopy and AFM. In the case of samples incubated in methyl α-mannoside solution, polymer film was found to remain on the surface, so the samples were returned to the solution and re-examined after 3 d, when surfaces free from polymer film were observed (Fig. S5).

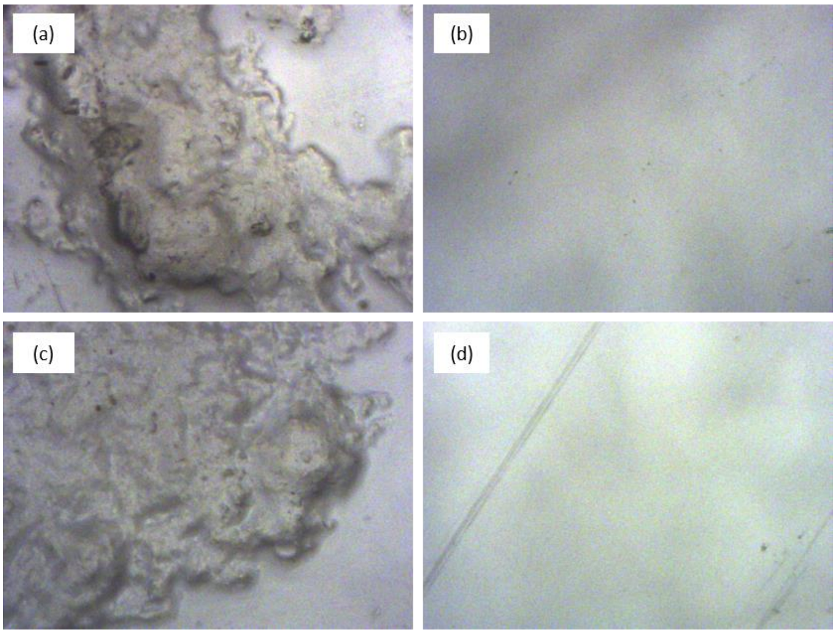

Fig. S5 Optical microscope images (40 X magnification) of (a) polymer film produced by exposure of **SCPN-MAN** to a Con A functionalised surface; (b) the same surface after exposure to NH_2OH (c) polymer film produced by exposure of **SCPN-MAN** to a Con A functionalised surface; (d) the same surface after exposure to methyl α-mannoside.

Film damage repair experiments

A polymer film of **SCPN1-MAN** on Con A functionalised Si wafer was generated as previously described, and scratched under PBS (pH 7.4) in a fluid cell using contact mode AFM by applying a voltage of 6.00 V (1.8 µN) in a 16 x 1 µm area, using an Bruker MPP-21100-10 tip where $k \approx 3$ N/m and $r \approx 8$ nm and the tip has no coating. The area were then imaged (also under PBS in contact mode) using a DNP – s10 tip where $k \approx 0.24$ N/m and $r \approx 20$ nm revealing a scratch across the surface (Fig. S6a,b)). The buffer was removed from the fluid cell and replaced with 100 mM NH_4OAc, pH 4.5. The sample was left in place for 24 h, then the area was re-examined by AFM (Fig. S6c,d), revealing a more uniform depth profile, suggesting that the polymer film had rearranged to 'heal' itself after damage.

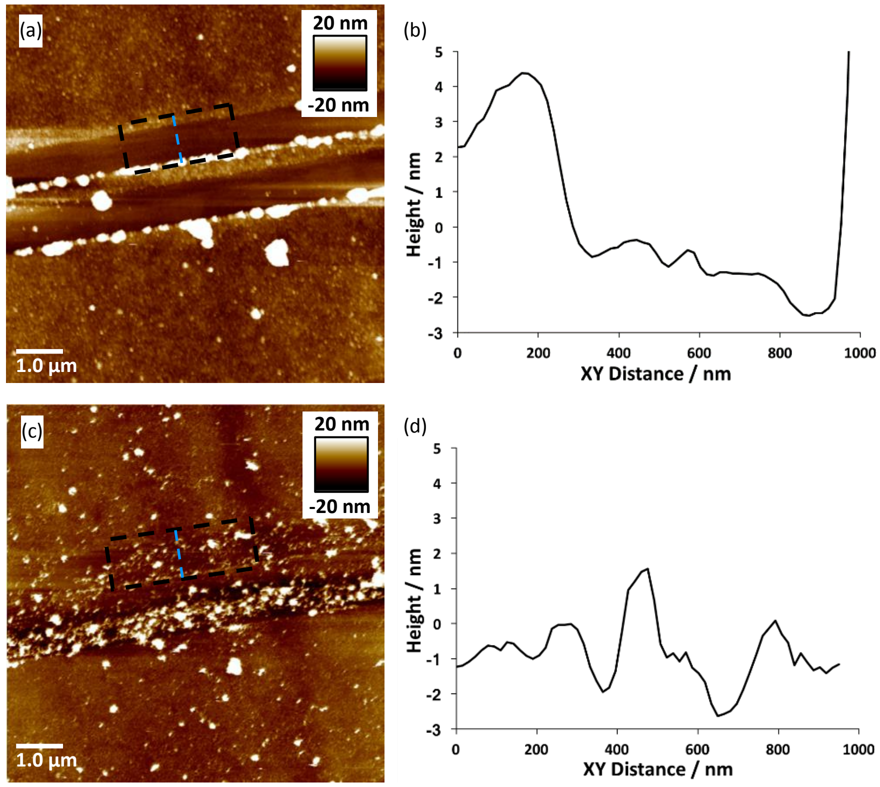

Fig. S6 Contact mode AFM images of a polymer film formed using **SCPN1-MAN** and Con A functionalised silicon wafer after being subjected to a scratch (a) and the corresponding height profile (b). (c) The same region after 18 h incubation in 100 mM NH_4OAc, pH 4.5 and the corresponding height profile, (d).

References

[1] C. S. Mahon, M. A. Fascione, C. Sakonsinsiri, T. E. McAllister, W. B. Turnbull, D. A. Fulton, *Org. Biomol. Chem.* **2015**, *13*, 2756-2761.

[2] R. Leece, T. R. Hirst, *Microbiology* **1992**, *138*, 719-724.

[3] J. Kim, J. Cho, P. M. Seidler, N. E. Kurland, V. K. Yadavalli, *Langmuir* **2010**, *26*, 2599-2608.
